# Conserved RNA secondary structure in *Cherry virus A* 5′-UTR associated with translation regulation

**DOI:** 10.1186/s12985-022-01824-z

**Published:** 2022-05-26

**Authors:** Deya Wang, Chen Yang, Yanmei Deng, Xue Cao, Wei Xu, Zishuo Han, Qingliang Li, Yang Yang, Xuefeng Yuan

**Affiliations:** 1 Department of Biotechnology, College of Life Sciences, Zaozhuang University, Zaozhuang, 277160 People’s Republic of China; 2 Department of Plant Pathology, College of Plant Protection, Shandong Agricultural University, Shandong Province Key Laboratory of Agricultural Microbiology, Tai’an, 271018 People’s Republic of China

**Keywords:** Translation elements, RNA structure, Evolution, *Cherry virus A*

## Abstract

**Background:**

A variety of *cis*-acting RNA elements with structures in the 5′- or 3′-untranslated region (UTR) of viral genomes play key roles in viral translation. *Cherry virus A* (CVA) is a member of the genus *Capillovirus* in the family *Betaflexiviridae*. It has a positive single-stranded RNA genome of ~ 7400 nucleotides (nt). The length of the CVA 5′-UTR is ~ 100 nt; however, the function of this long UTR has not yet been reported.

**Methods:**

Molecular and phylogenetic analyses were performed on 75 CVA sequences, which could be divided into four groups, and the RNA secondary structure was predicted in four CVA 5′-UTR types. These four CVA 5′-UTR types were then inserted upstream of the firefly luciferase reporter gene *FLuc* (*FLuc*), and in vitro translation of the corresponding transcripts was evaluated using wheat germ extract (WGE). Then, in-line structure probing was performed to reveal the conserved RNA structures in CVA-5′UTR.

**Results:**

The four CVA 5′-UTR types appeared to have a conserved RNA structure, and the *FLuc* construct containing these four CVA 5′-UTR types increased the translation of *FLuc* by 2–3 folds, suggesting weak translation enhancement activity. Mutations in CVA 5′-UTR suppressed translation, suggesting that the conserved RNA structure was important for function.

**Conclusion:**

The conserved RNA secondary structure was identified by structural evolution analysis of different CVA isolates and was found to regulate translation.

## Background

In viruses, the biological functions of RNA based on their spatial structures and various cis-acting RNA elements are translation, RNA synthesis, and genome packaging [1–4]. Efficient protein expression is a fundamental process in the life cycle of viruses, and translation elements with structures in the 5′- or 3′-untranslated region (UTR) of specific messenger RNAs are known to control expression [5–8]. Based on location and function, cis-acting elements are termed 5′ internal ribosome entry sites (5′ IRESs) and 3′ cap-independent translation elements (3′ CITEs). Both 5′ IRESs and 3′ CITEs are modular, functional RNA elements that can recruit ribosomes to stimulate cap-independent translation. Several classes of translation elements in viral genomes have been identified and described according to their well-defined secondary structures and distinct translation, initiation mechanisms [6, 9–11]. Despite being considerably diverse in sequence, most translation elements in viruses of different genera or even families have a conserved structure [12–14]. In particular, homologous structures could be important sequence motifs guiding efficient translation, but less studies have focused on the structural evolution of these translation elements among different isolates of given RNA virus [15]. In addition, such RNA structural elements may offer compelling targets for antivirals, thus laying the foundation for the treatment of plant viral diseases [16, 17].

Cherry virus A (CVA), a member of the genus *Capillovirus* in the family *Betaflexiviridae*, was first reported in 1995 [18]. CVA has a positive single-stranded RNA genome of ~ 7400 nucleotides (nt), excluding the poly(A) tail at its 3′ end, and encodes two open-reading frames (ORFs) [19, 20]. The length of the CVA 5′-UTR is ~ 100 nt, and the function of this long UTR has not yet been reported. In this study, molecular and phylogenetic analyses were performed on 75 CVA sequences, which could be divided into four groups. We predicted the RNA secondary structure, which appeared to be conserved, in four CVA 5′-UTR types. These four CVA 5′-UTR types were then inserted upstream of the firefly luciferase reporter gene *FLuc* (FLuc), and in vitro translation of the corresponding transcripts was evaluated using wheat germ extract (WGE), suggesting weak translation enhancement activity. This study identified a cis-acting element with conserved RNA secondary structure in CVA 5′-UTR, which presents IRES activity.

## Methods

### Virus material and genotyping

All CVA nucleotide sequences were referenced from the NCBI nucleotide database (http://www.ncbi.nlm.nih.gov/nucleotide/). The sequences were spliced into 20-bp reads using Perl scripts, and the reads were mapped against the NCBI reference sequence NC_003689.1 (https://www.ncbi.nlm.nih.gov/nuccore/NC_003689.1) using the “–best –strata -m 1” option in Bowtie. Single nucleotide polymorphism (SNP) detection was performed using the SAMtools and BCFtools pipeline ( http://htslib.org/ for the new 1.x releases of SAMtools, BCFtools, and HTSlib).

### Principal component analysis (PCA) and phylogenetic analyses

The population structure of the 75 variants was estimated by principal component analysis (PCA) using the PLINK 1.90 and EIGENSTRAT 6.1.4 programs. Genetic relationships were estimated using neighbor-joining trees constructed using the PHYLIP 3.695 software package. The VCF file was transformed to the PHYLIP 3.695 format using the Python script vcf2phylip (https://github.com/edgardomortiz/vcf2phylip).

### Nucleotide diversity

The nucleotide diversity of each group (π, the average number of nucleotide differences per site between two DNA sequences randomly chosen from the sample population) was calculated using VCFtools (https://sourceforge.net/projects/vcftools/files/) with “–window-pi 200 –window-pi-step 50”. The graph was plotted with R.

### Motif prediction

The motifs of the four CVA 5′-UTR types were predicted by the MEME online program (http://meme-suite.org/tools/meme) with the default option.

### RNA structure modeling

The RNA secondary structure of CVA was predicted with RNA Folding Form (version 2.3 energies) at the Mfold web server (http://unafold.rna.albany.edu/?q=mfold/RNA-Folding-Form2.3). The folding temperature was 25 °C, and the “other” default settings was used. All RNA structures were drawn using the online drawing tool RNA2Drawer (https://rna2drawer.app/).

### Plasmid construction and RNA preparation

Plasmids were constructed based on the *FLuc* reporter construct pT7-F-3-UTRssp vector by polymerase chain reaction amplification, enzyme digestion, and ligation. All nucleotide sequences of these plasmids were confirmed by DNA sequencing. All *FLuc* reporter constructs containing a CVA 5′-UTR sequence were linearized with *NruI* to create the template for RNA preparation. DNA fragments were amplified using PCR with a 5′primer that contained a T7 RNA polymerase promoter to be the template for preparing corresponding in vitro transcripts, which were used for in-line probing assays. RNA was transcribed in vitro using bacteriophage T7 RNA polymerase (Promega, USA) according to the manufacturer′s instructions. RNA integrity was estimated by 1.0% agarose gel electrophoresis, and RNA concentration was measured using a NanoDrop spectrophotometer.

### In vitro translation

In vitro translation assays were performed as previously described [15]. 3 pmol of RNA transcripts were used in a 25 μL translation reaction using wheat germ extract (WGE) (Promega) according to the manufacturer′s instructions. The luciferase activity was measured using a luciferase assay reporter system (Promega) and a Modulus Microplate Multimode Reader (Turner BioSystems). At least three independent in vitro translationa ssays were performed for each construct. Standard errors were calculated in Microsoft Excel.

### In-line structure probing

In-line probing was performed as previously described [10]. Briefly, RNA fragments of the CVA 5′-UTR (positions 1–106) was 5′end-labeled with [γ-^32^P] ATP, which was purified by electrophoresis through 5% denaturing polyacrylamide gels. End-labeled RNA denatured at 75 °C, followed by slowly cooling to 25 °C. For in-line probing, 5 pmol of end-labeled RNA was incubated at 25 °C in 50 mM Tris–HCl (pH 8.5) and 20 mM MgCl_2_ for 12 h. Samples were separated by 8% polyacrylamide gel electrophoresis (8 M urea) alongside a hydroxide-generated RNA cleavage ladder and RNase T1 digestion product on labeled RNA fragments. Then, the gels were dried and exposed to a phosphorimager screen, followed by detection with the Typhoon FLA 7000 (GE Healthcare).

## Results

### Population characterization

**Fig. 1:**
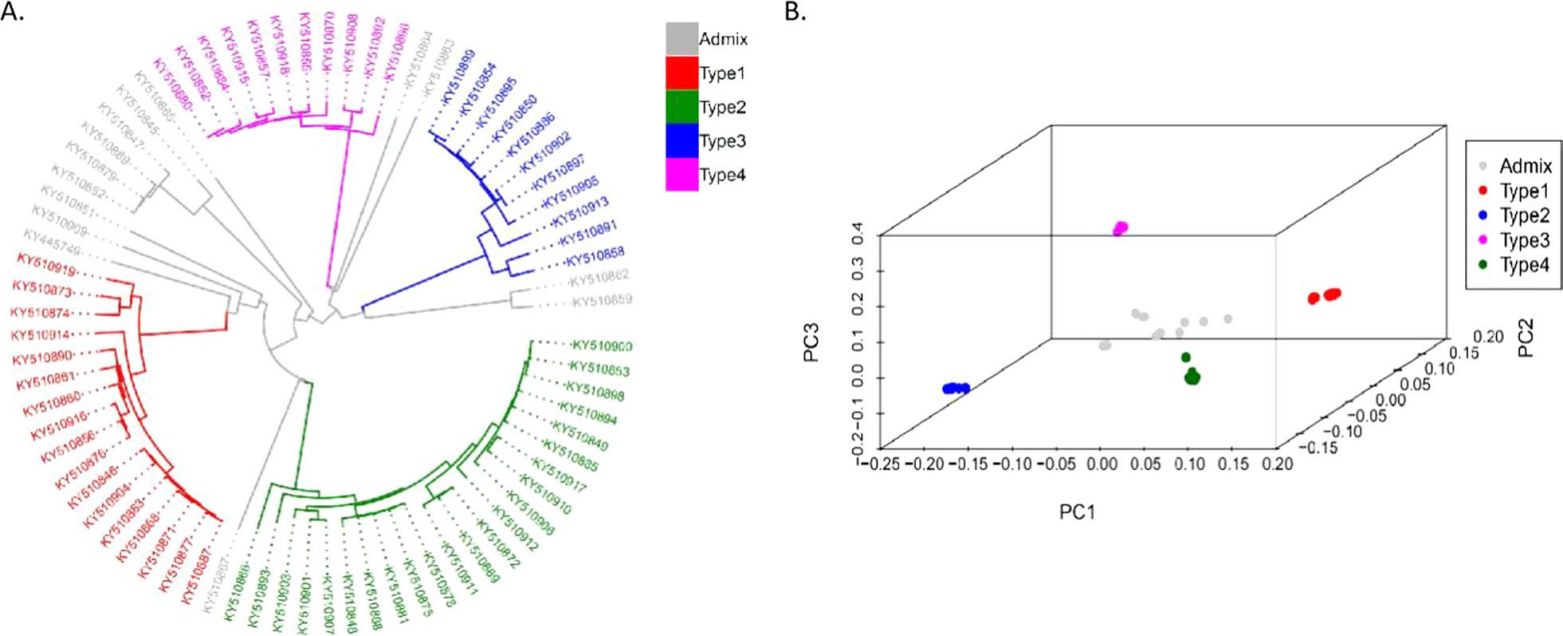
Genetic structure and diversity of CVA. **A** Neighbor-joining phylogenetic tree of 75 CVA variants. **B** 3D PCA (PC1, PC2, and PC3) of 75 CVA variants using identified SNPs as markers. CVA variants from the same subgroup clustered together

To effectively investigate the CVA RNA structure, a population that was neither genetically highly structured nor interrelated was selected. Using SNPs (single nucleotide polymorphism, SNP), principal component analysis (PCA) was performed and a phylogenetic tree was constructed to quantify the population structure of the 75 variants. On the basis of the phylogenetic tree and PCA analyses, five clades were classified—types 1–4 and Admix (Fig. 1). In the phylogenetic tree, the Admix clade was composed of disordered variants, but the CVA types 1–4 were distinct. Thus, the four CVA types were used for further analysis. Types 1 and 2 were on two near branches and were the same as types 3 and 4, indicating that types 1 and 2 had a genetic model similar to types 3 and 4.

### Nucleotide diversity in different groups

**Fig. 2:**
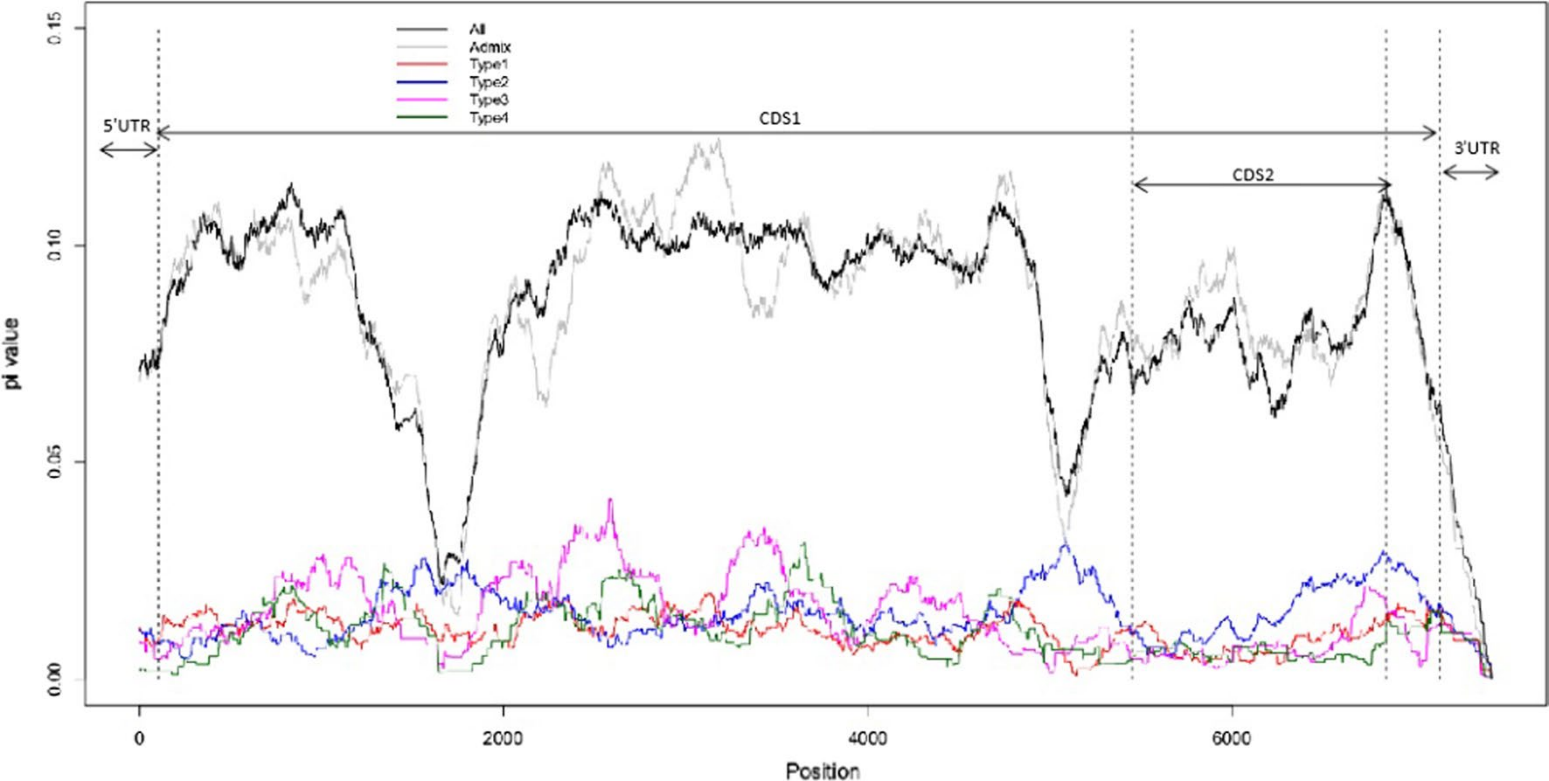
Nucleotide diversity in different groups on the whole genome. Polyline height represents nucleotide diversity, and the x-axis represents genome position. Dotted lines divide the genome into different functional regions

The fine-scale maps for nucleotide diversity (π) of the four types and all CVAs showed great variation along the whole genome (Fig. 2). The π values of all CVAs were strikingly different from the four types, indicating that the CVA classification was reasonable. Some regions had a higher π value in one type (mainly types 2 and 3) than in the other three types. These regions, particularly 5′-UTR and 3′-UTR, showed induced mutations in a certain type. The π values of 5′- and 3′-UTRs were low in all types. The initial sequence of CDS2 had the same state. Therefore, these three regions were highly conserved, implying that they played important functions.

### Diverse motifs of the four CVA 5′-UTR types

**Fig. 3:**
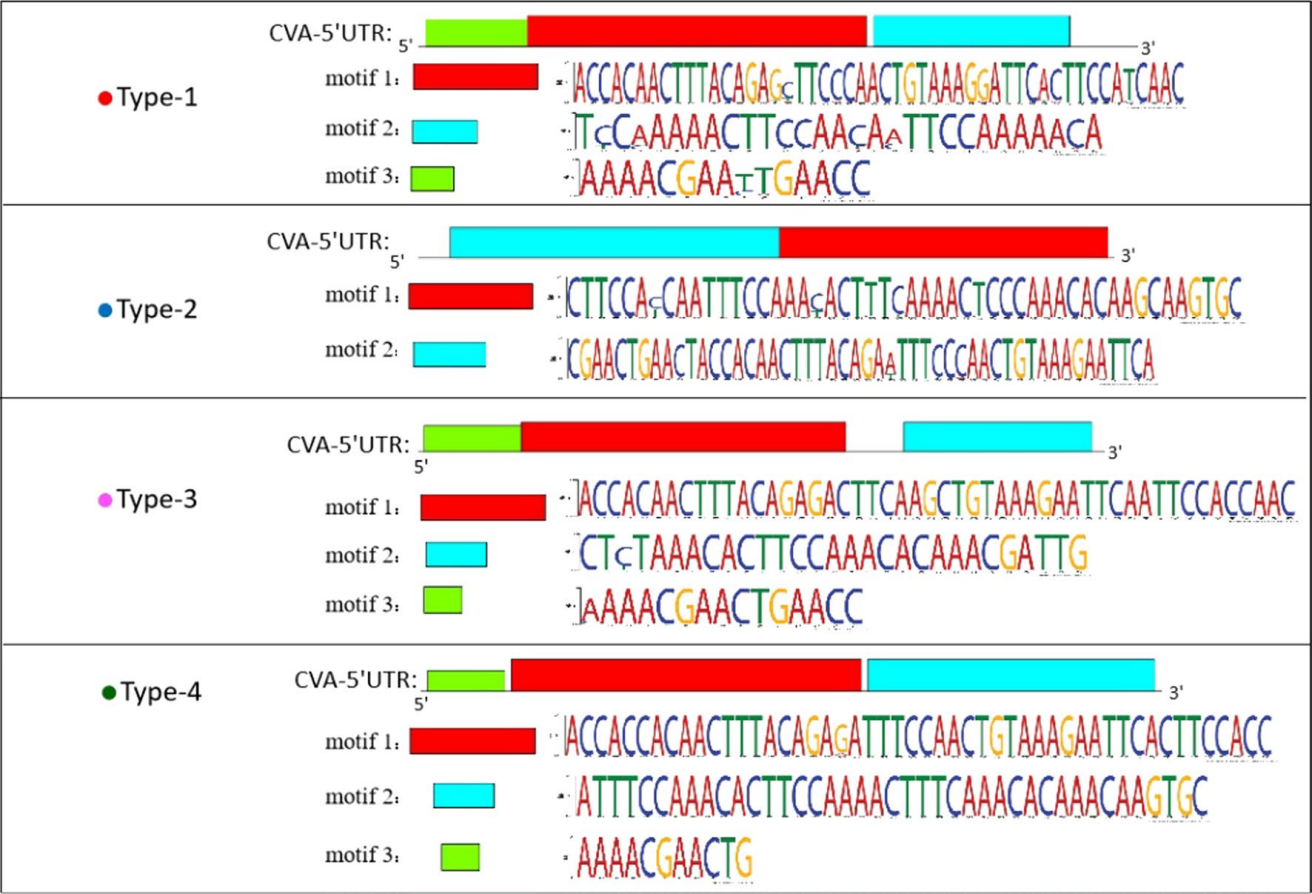
Motifs in the four CVA 5′-UTR types. Similar motifs are marked in red, and different motifs are marked in turquoise. The red motif sequence is more conservative, and the turquoise motif sequence is the hypervariable region. Green motifs were absent in type 2

Gene expression was possibility controlled by the 5′-UTR structure determined by nucleotide sequence. The MEME program was used to predict the motifs of the 5′-UTR sequences in the four types (Fig. 3). Almost all motif sites were one base, and the frequency of the base was 100%. There were three motifs in types 1, 3, and 4, and two motifs in type 2. The structures of the four CVA 5′-UTR types comprised different motifs, and differences in the four types induced different structures.

### Secondary structures in the four CVA 5′-UTR types

**Fig. 4:**
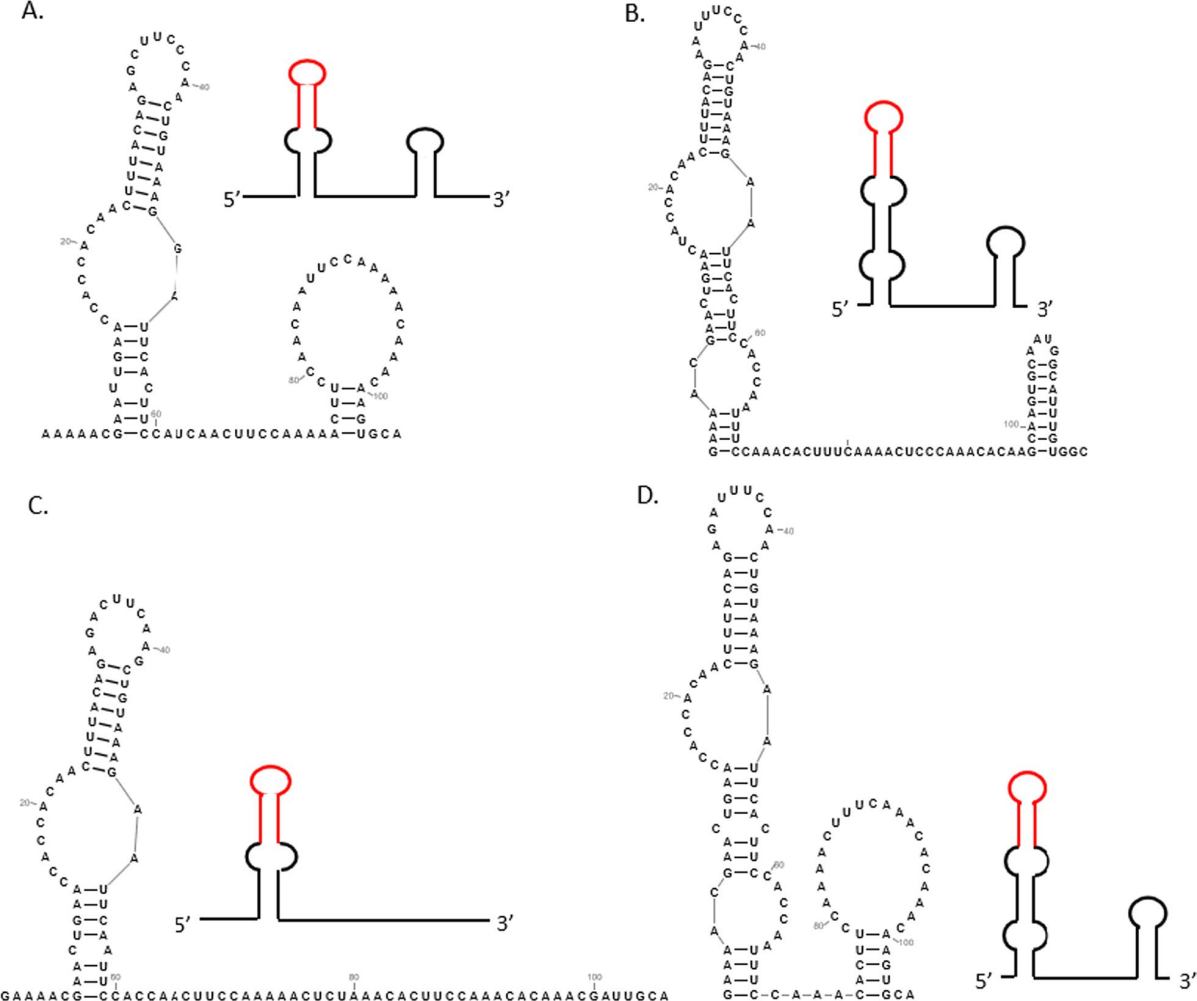
Secondary structures in the four CVA 5′-UTR types. A-D correspond to 5′-UTR types 1–4, respectively. The red line indicates the conserved RNA structure

The RNA secondary structures were predicted through the conserved motif sequences using the Mfold software (Fig. 4). Type 1, 2, and 4 structures were formed by two stem-loops, the type 3 structure formed by only one stem-loop, but they showed evidence for phylogenetically conserved terminal stem-loops. The sequences of the common loop that probably recruit ribosomes were dissected, and their compositions were slightly different. On analyzing the characteristics of the secondary structures of 5′-UTR in the four CVA types, CVA 5′-UTR was found to contain a conserved sequence and RNA structure region (CR), CUUUACAGAGCUUCCCAACUGUAAAG, that forms a stem-loop in which the under lined bases are paired. And its loop is particularly rich in pyrimidine. Thus we hypothesized that the polypyrimidine sequences could interact with a region of 18S rRNA to enhance translation activity, as was previously found for *Tomato bushy stunt virus, Barley yellow dwarf virus, Turnip crinkle virus and Triticum Mosaic Virus* [21–23].

### Translation enhancement activity of CVA 5′-UTR

**Fig. 5:**
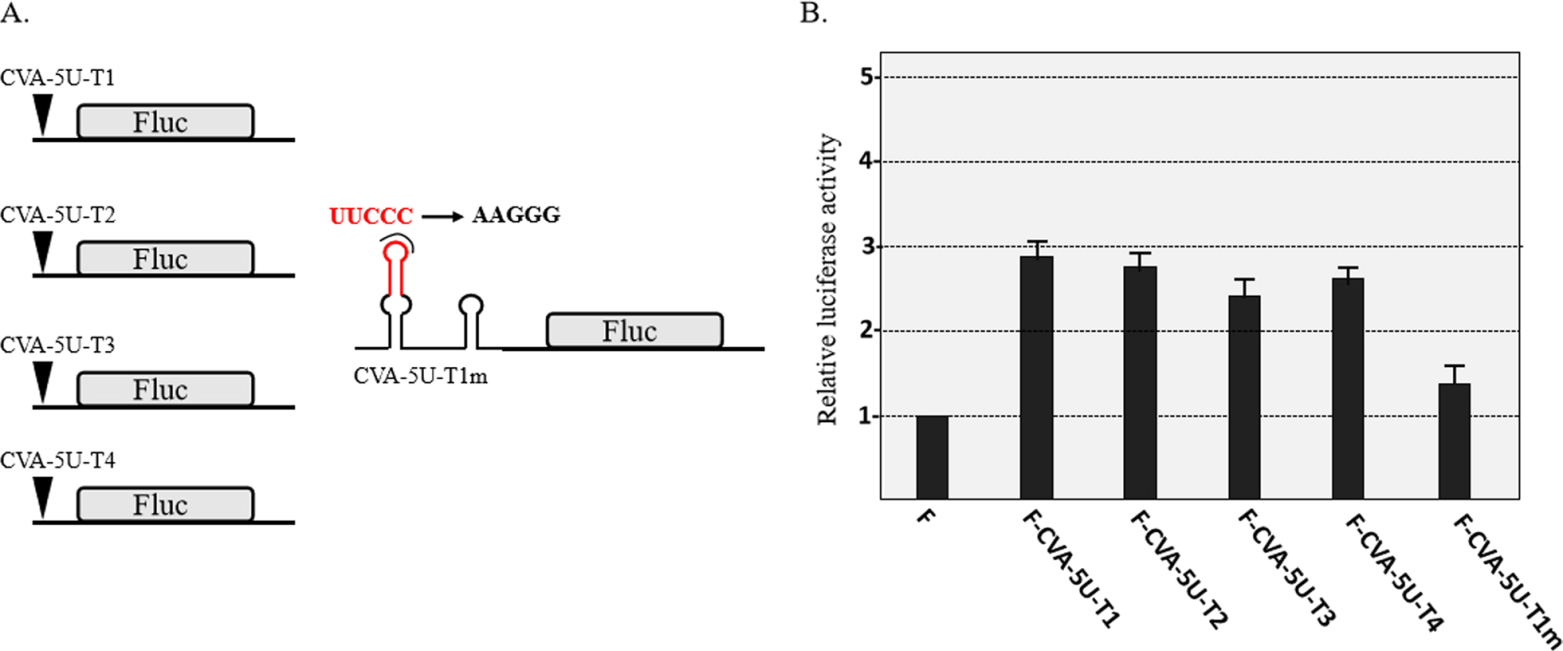
Effects of CVA 5′-UTR types and mutation of CR on translation of the *FLuc* reporter gene. **A** *FLuc* reporter constructs containing CVA 5′-UTR types. Red nucleotides indicate the mutation sites. **B** Effects of CVA 5′-UTR types and mutation of CR on *FLuc* translation

To evaluate the effects on translation, the four CVA 5′-UTR types were inserted upstream of the *FLuc* gene (Fig. 5A), and in vitro translation of the corresponding transcripts was evaluated using WGE. These four 5′-UTR types enhanced the translation of *FLuc* by 2–3 folds in the absence of the 5′cap (Fig. 5B), which showed cap-independent translation enhancement activity. In addition, the effect of conserved RNA structure on translation was analyzed through mutagenesis of the *FLuc* reporter constructs (Fig. 5A). Results show that for the loop of CR, the mutation of U^35^U^36^C^37^C^38^C^39^ to AAGGG reduced the translation to 47% of that of F-CVA-5U-T1 (Fig. 5B). However, the mutation didnot reduce translation to a level comparable to that of the *FLuc,* that may be because CVA 5′-UTR has some polypyrimidine-rich repeats.

### Structure solution probing of CVA-5′UTR-type 1

**Fig. 6:**
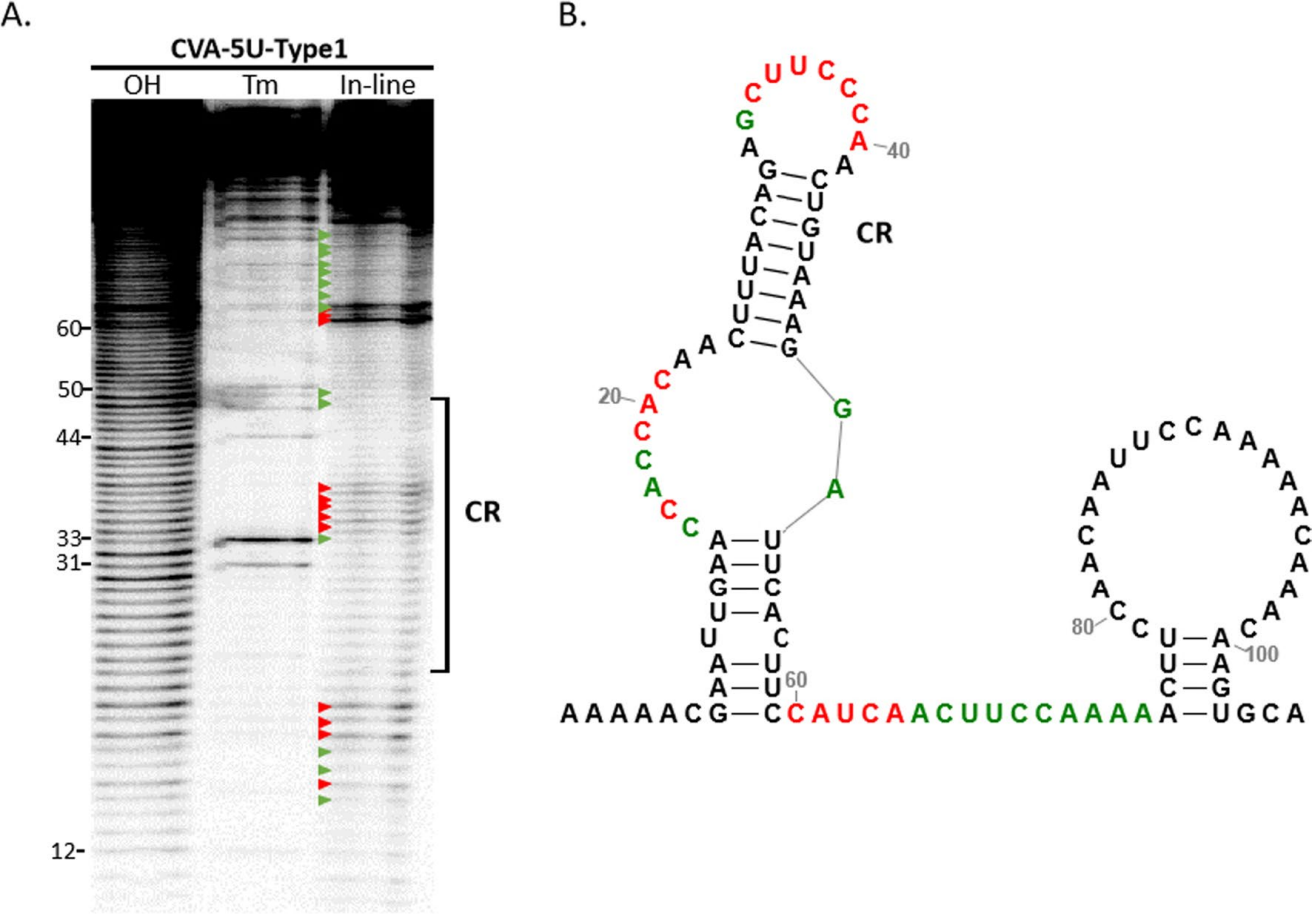
In-line structure probing of CVA 5′-UTR-Type-1. **A** Susceptibility of residues at the CVA 5′-UTR-Type-1 to in-line cleavage. OH, ladder generated from treatment with NaOH; Tm, partial RNaseT1 digest to denote location of guanosine; In-line, in-line cleavage of the fragment. The intensity of each band is proportional to the flexibility of the residue at that location. Green solid triangles point to the weaker cleavage sites, where as red solid triangles point to the stronger cleavage sites. CR, conserved sequence and RNA structure region. **B** Susceptibility of residues in the putative structure of CVA 5′-UTR-Type-1 to in-line cleavage. Green and red nucleotides indicate the single-strand characteristic, whereas red nucleotides indicate strongly marked cleavage based on the in-line probing pattern

Although the secondary structures of CVA 5′-UTR have been predicted, we set out to evaluate the conserved RNA structure directly. Owing to the CVA 5′-UTR regions were highly conserved, we select type 1 to identify the conserved RNA structure by in line probing assay. In-line probing reports on the spontaneous cleavage of the RNA backbone mediated by 2′-hydroxyl that are geometrically in-line with oxyanion leaving groups on backbone phosphates. Such in-line geometry occurs primarily in nonstructured regions of RNA, where nucleotides are not torsionally constrained by hydrogen bond pairing. Based on Mfold and in-line cleavage pattern, CVA-5′-UTR-type 1 has two hairpins (Fig. 6B). Most residues on the loop of CR were more susceptible to cleavage, and it showed that the conserved sequence region contains a stable stem-loop (Fig. 6A, B).

## Discussion

Sweet cherry is a major fruit crop of increasing economic importance. CVA is among the most common viruses infecting sweet cherry [24, 25]. In some regions of China, the CVA detectable rate in sweet cherry leaf samples is high, up to ~ 60% [25]. Molecular evolution studies on CVA can help us to understand the important features of RNA viruses, such as the population structure and the underlying evolutionary mechanisms. Several such studies have recently been published. Gao et al*.* (2017) reported the genetic diversity of CVA isolates from China by analyzing three genomic regions that corresponded to the coat protein, RNA-dependent RNA polymerase, and the core region, which resulted in at least seven major clusters [19]. Moreover, 75 full-length CVA sequences were assembled from next-generation sequencing data by Kesanakurti et al*.* (2017), Phylogenetic analysis resulted in six major groupings [26]. In our study, the genetic diversity and evolution of 75 sequences were further analyzed using PCA and phylogenetic tree construction, and five clades were classified—types 1–4 and Admix (Fig. 1). Nucleotide diversity analysis in different groups showed that the UTR sequence is conserved, indicating that it may have regulation and control functions.

Conserved stem-loop structures that function as translational enhancers have been previously identified in viruses [27–29]. To date, several types of translational enhancers have been reported in different RNA viruses possessing different structural characteristics [10, 11, 30, 31]. IRESs are unique RNA elements, which use stable and dynamic RNA structures to recruit ribosomes and drive protein synthesis. However, some IRESes did not present a remarkable secondary structure. This IRES activity requires a Watson–Crick base-pairing interaction between the IRES and 18S rRNA of 40S ribosomal subunits, which has been found in RNA2 of the blackcurrant conversion virus (BRV). In this study, the RNA structure of CVA 5′-UTR was first identified by combining phylogenetic and predictive strategies, and CVA 5′-UTR was found to regulate translation. According to the structural evolution analysis, the RNA structure of CVA 5′-UTR was conserved, the loop of which is particularly rich in pyrimidine. And it suggests that the polypyrimidine sequences could interact with a region of 18S rRNA to enhance translation activity [21–23]. In addition, a similar model has been found in eukaryotic cells. Indeed, UTRs and most noncoding RNAs produced in eukaryotes are either not conserved or so highly conserved that they have many biological functions [7]. Although several conserved secondary structure motifs in viral genomes have been identified by alignment-based structure prediction, the underlying structural evolutionary mechanism remains unclear.

## Conclusion

Molecular and phylogenetic analyses were performed on 75 CVA sequences, which could be divided into four groups. The four CVA 5′-UTR types appeared to have a conserved RNA structure, and the *FLuc* construct containing these CVA 5′-UTR types increased the translation of *FLuc* by 2–3 folds, suggesting weak translation enhancement activity. We identified the conserved RNA structure by structural evolution analysis of different CVA isolates and found that it presents IRES activity to regulate translation.

## Data Availability

All CVA nucleotide sequences were referenced from the NCBI nucleotide database (http://www.ncbi.nlm.nih.gov/nucleotide/).

## Abbreviations

UTR: Untranslated region
CVA: *Cherry* *virus** A*
nt: Nucleotide
FLuc: Firefly luciferase
WGE: Wheat germ extract
5′ IRES: 5′ Internal ribosome entry sites
3′ CITE: 3′ Cap-independent translation element
PCA: Principal component analysis
SNP: Single nucleotide polymorphism
CR: Conserved region
